# Chemotaxonomic Metabolite Profiling of 62 Indigenous Plant Species and Its Correlation with Bioactivities

**DOI:** 10.3390/molecules201119652

**Published:** 2015-11-02

**Authors:** Sarah Lee, Dong-Gu Oh, Sunmin Lee, Ga Ryun Kim, Jong Seok Lee, Youn Kyoung Son, Chang-Hwan Bae, Joohong Yeo, Choong Hwan Lee

**Affiliations:** 1 National Institute of Biological Resources, Environmental Research Complex, Incheon 404-708, Korea; lsr57@korea.kr (S.L.); ryun31@korea.kr (G.R.K.); jslee001@korea.kr (J.S.L.); sophy004@korea.kr (Y.K.S.); bae0072@korea.kr (C.-H.B.); y1208@korea.kr (J.Y.); 2 Department of Bioscience and Biotechnology, Konkuk University, Seoul 143-701, Korea; dhehdrn@konkuk.ac.kr (D.-G.O.); duly123@naver.com (S.L.)

**Keywords:** chemotaxonomy, indigenous plant, metabolite profiling, UHPLC-LTQ-IT-MS/MS, antioxidant activity, tyrosinase inhibition activity

## Abstract

Chemotaxonomic metabolite profiling of 62 indigenous Korean plant species was performed by ultrahigh performance liquid chromatography (UHPLC)-linear trap quadrupole-ion trap (LTQ-IT) mass spectrometry/mass spectrometry (MS/MS) combined with multivariate statistical analysis. In partial least squares discriminant analysis (PLS-DA), the 62 species clustered depending on their phylogenetic family, in particular, Aceraceae, Betulaceae, and Fagaceae were distinguished from Rosaceae, Fabaceae, and Asteraceae. Quinic acid, gallic acid, quercetin, quercetin derivatives, kaempferol, and kaempferol derivatives were identified as family-specific metabolites, and were found in relatively high concentrations in Aceraceae, Betulaceae, and Fagaceae. Fagaceae and Asteraceae were selected based on results of PLS-DA and bioactivities to determine the correlation between metabolic differences among plant families and bioactivities. Quinic acid, quercetin, kaempferol, quercetin derivatives, and kaempferol derivatives were found in higher concentrations in Fagaceae than in Asteraceae, and were positively correlated with antioxidant and tyrosinase inhibition activities. These results suggest that metabolite profiling was a useful tool for finding the different metabolic states of each plant family and understanding the correlation between metabolites and bioactivities in accordance with plant family.

## 1. Introduction

Because of its location and temperate climate, Korea has a wide diversity of plant species [1,2]. These various plant species are characterized by different compositions and amounts of the phytochemicals responsible for color and bioactive properties according to environmental factors such as water utility, temperature, climate, and cultivation period [3,4,5]. Some of these plant species contain beneficial secondary metabolic compounds, which contribute to bioactivities such as antioxidant, anti-inflammatory, antibacterial, and tyrosinase inhibitory activity [6,7,8]. Because of their bioactive utility, many indigenous Korean plants have been used for medical and other purposes, such as health promoting foods [9], anti-obesity medication [10], antioxidant and anticancer agents [11,12], and cosmetics [13]. To understand and effectively utilize the indigenous Korean plant species, taxonomic classification is necessary. Plant classification can be accomplished by comparing differences in properties of plant species, such as morphological [14], physiological [15], and chemical characteristics [16]. Among the various plant taxonomic methods, chemotaxonomy, a method based on differences in chemical compounds, is a useful tool for classification of plant species. Chemotaxonomic plant classification has been used to classify plant species according to their phylogenetic genus [17].

Recently, metabolomics has been used, and is a powerful tool for metabolite analysis such as quality control for food [18], metabolism of microorganisms [19], and human disease biomarkers [20]. Metabolomics is also a valuable tool for comprehensive identification and quantification of metabolites in plants, including plant metabolite profiling [21], analysis of plant compounds in food and medicine [18,22], and research of plant development [23]. For several decades, metabolomics has been used as a chemotaxonomic tool for classification of plant species [24]. Metabolomics based on liquid chromatographic separation combined with mass spectrometry offers detailed information on plant metabolites [25] and could be advantageous in botanical chemotaxonomy.

Metabolomics has been previously used in plant research. Liquid chromatography-mass spectrometry (LC-MS)-based metabolite profiling is valuable for analysis of compounds over a wide range of polarity and molecular weight [26]. Beneficial secondary compounds in plants, such as flavonoids, phenolic compounds, and terpenoids have been identified [27,28], and plant bioactivities, including antioxidant activity [29], antimicrobial activity, and tyrosinase inhibition activity [30] have been reported. However, few studies have attempted to reveal the relationship between metabolite differences and bioactivity in diverse plant species. In this study, metabolite profiling of 62 indigenous Korean plant species, in 6 phylogenetically distant botanical families (Aceraceae, Betulaceae, Fagaceae, Rosaceae, Asteraceae, and Fabaceae), was performed using LC-MS for chemotaxonomic classification. In addition, we selected significantly different metabolites among plant families and analyzed their correlation with bioactivity.

## 2. Results and Discussion

### 2.1. Chemotaxonomic Metabolite Profiling of 62 Indigenous Korean Plant Species

**Figure 1:**
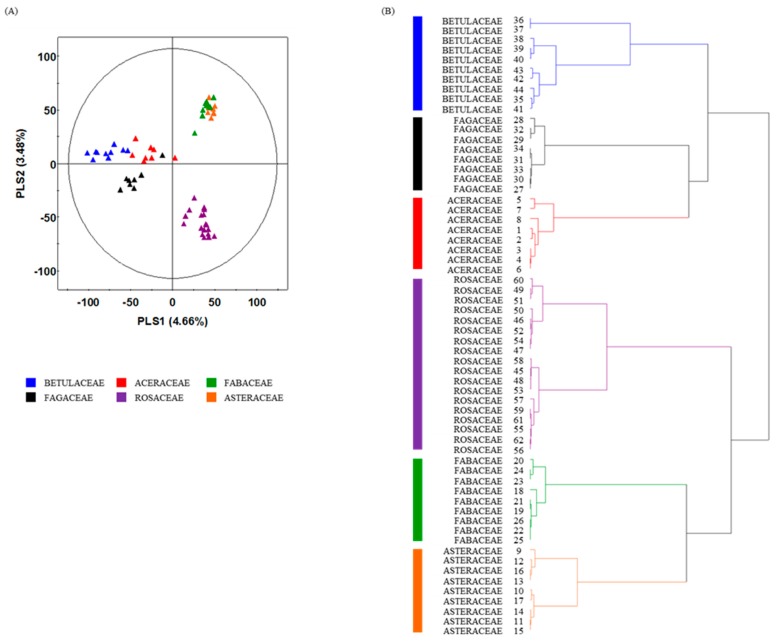
Partial least squares discriminant analysis (PLS-DA) score plot (**A**) and hierarchical cluster analysis (HCA) dendrogram based on PLS-DA results (**B**) derived from the ultrahigh performance liquid chromatography (UHPLC)-linear trap quadrupole-ion trap (LTQ-IT) mass spectrometry/mass spectrometry (MS/MS) data of 62 indigenous Korean plant species. Samples are colored according to the family.

**Figure 2:**
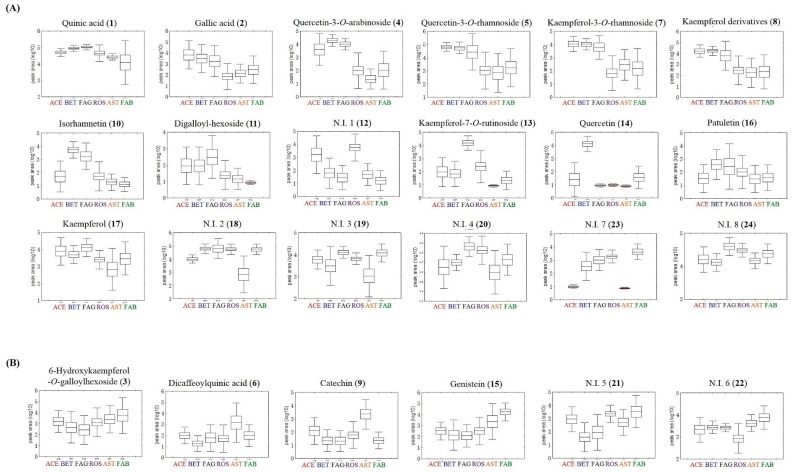
Box and whisker plots of significantly different metabolites among 6 plant families analyzed by ultrahigh performance liquid chromatography (UHPLC)-linear trap quadrupole-ion trap (LTQ-IT) mass spectrometry/mass spectrometry (MS/MS). Metabolites at higher concentrations in Aceraceae, Betulaceae, and Fagaceae (**A**) and at higher concentrations in Rosaceae, Asteraceae, and Fabaceae (**B**) are shown. The *Y*-axis of the box and whisker plots indicates the peak area of each metabolite transformed by log_10_ (ACE, Aceraceae; BET, Betulaceae; FAG, Fagaceae; ROS, Rosaceae; AST, Asteraceae; FAB, Fabaceae; *Line*, mean; *box*, standard error; *whisker*, standard deviation).

Sixty-two indigenous Korean plant species were analyzed by ultrahigh performance liquid chromatography (UHPLC)-linear trap quadrupole-ion trap (LTQ-IT) mass spectrometry/mass spectrometry (MS/MS) combined with multivariate statistical analysis. Metabolite profiling was used as a chemotaxonomic tool for analyzing differences in metabolites among the 62 plant species. In principal component analysis (PCA) and partial least squares discriminant analysis (PLS-DA), the 62 species were clustered depending on their phylogeny (Figure 1A, Figure S1A). Betulaceae, Fagaceae, and Aceraceae clusters were distinguished from Asteraceae, Fabaceae, and Rosaceae clusters by PLS1 (4.66%). The feature values were a metabolic data set analyzed by UHPLC-LTQ-IT-MS/MS and class information was plant phylogeny, in particular, family. PLS-DA clearly showed clustering patterns (Figure 1A) because it used class information in addition to feature values, which helped to determine whether the species were correctly classified. Hierarchical cluster analysis (HCA) dendrograms based on PCA (Figure S1B) and PLS-DA results (Figure 1B) derived from the UHPLC-LTQ-IT-MS/MS dataset showed merging patterns by each plant family. There were two large groups; one consisted of the Betulaceae, Fagaceae, and Aceraceae, and the other was composed of the Rosaceae, Fabaceae, and Asteraceae. Although samples were collected from different areas at various times, multivariate statistical analysis indicated that metabolic differences in plant species mainly depended on phylogenetic properties rather than environmental factors. Similar research revealed that differences in secondary metabolites of plants were affected by species rather than geological difference [31]. Twenty-four metabolites were considered as significantly different metabolites among the 6 plant families by variable importance in the projection (VIP) > 0.7 and *p*-value < 0.05 (Table 1). Sixteen metabolites were tentatively identified by comparing mass spectra and retention time of standard compounds or mass to charge ratio, mass fragment patterns, and UV absorbance according to references [32,33,34,35,36]. The identified metabolites were polyols (quinic acid and dicaffeoylquinic acid), phenolic compounds (gallic acid and digalloyl-hexoside), and flavonoids and flavonoid derivatives (quercetin, quercetin derivatives, kaempferol, kaempferol derivatives, isorhamnetin, patuletin, catechin, and genistein), which are known secondary metabolic compounds in various plant species [32,33,34,35,36]. Including eight non-identified metabolites, relative amounts of the 24 metabolites among the six families are shown in doughnut charts (Figure S2) and the box and whisker plots (Figure 2). Quinic acid (**1**), gallic acid (**2**), quercetin-3-*O*-arabinoside (**4**), quercetin-3-*O*-rhamnoside (**5**), kaempferol-3-*O*-rhamnoside (**7**), kaempferol derivatives (**8**), isorhamnetin (**10**), digalloyl hexoside (**11**), kaempferol-7-*O*-rutinoside (**13**), quercetin (**14**), patuletin (**16**), kaempferol (**17**), and non-identified (N.I.) metabolites (**12**, **18**–**20**, **23**–**24**) were at high concentrations in Aceraceae, Betulaceae, and Fagaceae, whereas 6-hydroxykaempferol-*O*-galloylhexoside (**3**), dicaffeoylquinic acid (**6**), catechin (**9**), genistein (**15**), and N.I. metabolites (**21**, **22**) were high in Rosaceae, Asteraceae, and Fabaceae. As shown in the box and whisker plots, the amounts of several metabolites were high in some families. Levels of isorhamnetin and quercetin were higher in Betulaceae than in other families. Isorhamnetin and quercetin are classified as flavonols, which were used as a chemotaxonomic marker for Betulaceae [37]. Levels of isorhamnetin and kaempferol-7-*O*-rutinoside were higher in Fagaceae. These substances have been detected in Fagaceae in other studies [32]. Catechin could be used as a marker for Asteraceae because of its high concentration. Previous studies highlighted catechin as an antioxidant phenolic compound in this family [38]. Genistein is a major isoflavone in the Fabaceae family [39] and exhibited the highest concentration in this family. Thus, genistein could be used as a marker for the family Fabaceae. These results indicated that the indigenous Korean plant species showed dissimilar metabolic states in accordance with plant phylogeny, which contributed to the grouping and separation patterns by family through multivariate statistical analysis. Moreover, some metabolites could be used as markers for a plant family because of the high concentration of metabolites in that family.

**Table 1:** Tentatively identified metabolites in 6 plant families that contributed to family clusters by PLS-DA.

No.	Putative Identification ^a^	RT ^b^ (min)	UHPLC-LTQXL-IT-MS/MS					*p*-Value	Id ^g^
			*m*/*z* Posi ^c^	*m*/*z* Nega ^d^	M.W. ^e^	MS^n^ Fragment Pattern ^f^	UV (nm)		
1	Quinic acid	1.02	-	191	192	173	214, 279	9.00E-04	Ref. [33]
2	Gallic acid	1.37	-	169	170	-	226, 272	3.00E-04	STD
3	6-Hydroxykaempferol-*O*-galloylhexoside	7.09	617	615	616	463 > 301	221, 265, 352	2.00E-04	Ref. [32]
4	Quercetin-3-*O*-arabinoside	7.89	435	433	434	301	239, 368	8.00E-06	Ref. [35]
5	Quercetin-3-*O*-rhamnoside	8.08	449	447	448	301 > 151	232, 277	4.00E-06	Ref. [35]
6	Dicaffeoylquinic acid	8.28	517	515	516	353 > 191	214, 322	4.90E-03	Ref. [33]
7	Kaempferol-3-*O*-rhamnoside	8.61	433	431	432	285	228, 280	3.40E-03	Ref. [35]
8	Kaempferol derivatives	8.63	479	477	478	431 > 285	271, 281	4.60E-03	Ref. [35]
9	Catechin	8.72	291	289	290	245	213, 303	2.90E-03	Ref. [36]
10	Isorhamnetin	8.74	317	315	316	300	366	4.00E-04	Ref. [32]
11	Digalloyl-hexoside	8.8	485	483	484	169	276	2.29E-02	Ref. [34]
12	N.I. 1	8.96	601	599	600	301 > 151	214, 268	1.60E-03	
13	Kaempferol-7-*O*-rutinoside	9.34	595	593	594	447 > 285	276	2.00E-09	Ref. [33]
14	Quercetin	9.69	303	301	302	151	214, 274	7.00E-04	STD
15	Genistein	10.47	271	269	270	253, 243, 215, 153	285, 318	3.70E-03	STD
16	Patuletin	10.59	333	331	332	287	269, 316	8.40E-03	Ref. [32]
17	Kaempferol	10.63	287	285	286	-	279	4.21E-02	STD
18	N.I. 2	10.77	229	227	228	165 > 111	202, 280	7.00E-04	
19	N.I. 3	10.88	329	327	328	197, 291	202, 279	3.00E-04	
20	N.I. 4	11.95	309	307	308	289 > 271	206, 282, 314	1.57E-02	
21	N.I. 5	12.97	313	311	312	293 > 275	218, 366	1.35E-02	
22	N.I. 6	13.78	315	313	314	201	221, 279	5.00E-04	
23	N.I. 7	14.53	529	527	528	277 > 233	226, 280	7.00E-04	
24	N.I. 8	14.57	295	293	294	275, 195	226, 280	3.80E-03	

^a^ Putative metabolites based on variable importance projection (VIP) analysis with cutoff value of 0.7 and a *p*-value <0.05. ^b^ Retention time. ^c^ Molecular ion detected in positive mode, [M + H]^+^. ^d^ Molecular ion detected in negative mode, [M − H]^−^. ^e^ Molecular weight. ^f^ MS^n^ fragment patterns detected in negative ion mode. ^g^ Identification: STD, standard compound/Ref., references.

**Figure 3:**
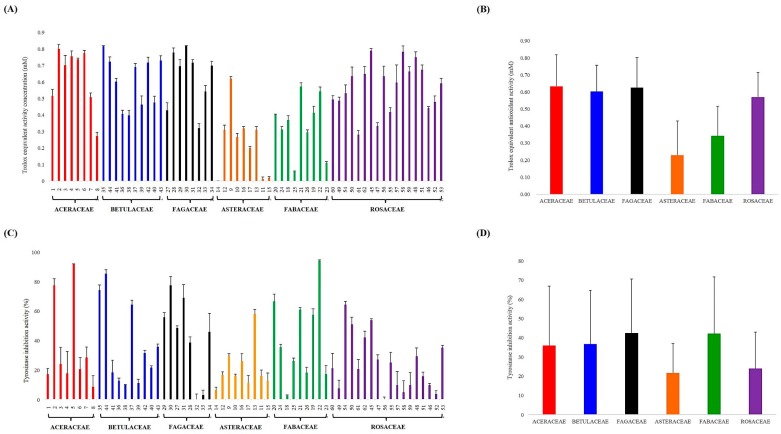
Antioxidant activity using DPPH radical scavenging assay (**A**) and tyrosinase inhibition activity (**C**) of 62 indigenous plant species, and average values of antioxidant (**B**) and tyrosinase inhibition activity (**D**) in each family.

### 2.2. Bioactivities of 62 Indigenous Korean Plant Species

To compare the bioactivities of the 62 indigenous Korean plant species, antioxidant activity using the DPPH radical scavenging assay and tyrosinase inhibition activity were measured. The antioxidant activity of 62 species (Figure 3A) and average values for each family (Figure 3B) are shown as the standard of Trolox equivalent antioxidant activity. Although there were variations in antioxidant activity among the species belonging to the same family, average values of antioxidant activity in order from highest to lowest were: Aceraceae, Fagaceae, Betulaceae, Rosaceae, Fabaceae, and Asteraceae. In Figure 3, tyrosinase inhibition activity of the 62 species (Figure 3C) and average value for each family (Figure 3D) are shown. The average values of tyrosinase inhibition activity in order from highest to lowest were: Fabaceae, Fagaceae, Betulaceae, Aceraceae, Rosaceae, and Asteraceae. However, wide individual variation in tyrosinase inhibition activity among the plant species in the same family was observed. Aceraceae, Betulaceae, and Fagaceae exhibited higher bioactivity than Asteraceae, Fabaceae, and Rosaceae (Figure 3B,D).

### 2.3. Correlation of Metabolic Differences and Bioactivities in Plant Families

**Figure 4:**
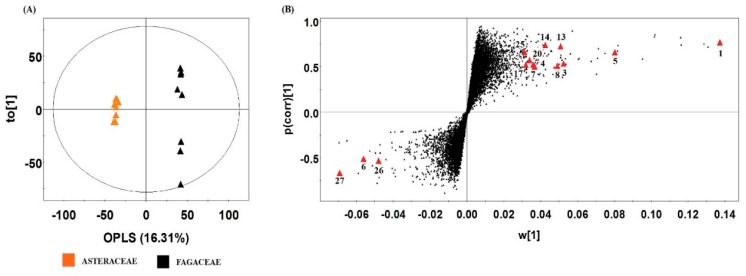
Orthogonal partial least squares discriminant analysis (OPLS-DA) score plot (**A**) and loading S-plot (**B**) derived from the ultrahigh performance liquid chromatography (UHPLC)-linear trap quadrupole-ion trap (LTQ-IT) mass spectrometry/mass spectrometry (MS/MS) dataset for Fagaceae and Asteraceae: R^2^X(0.281), R^2^Y(0.999), and Q^2^(0.854); R^2^X is all the Xs explained by the component, R^2^Y is all the Ys explained by the component, and Q^2^ is total variation of the Xs and Ys that can be predicted by the component. The significantly different metabolites (*p*-value < 0.05) are highlighted in the S-plot. The numbers indicated on the loading S-plot are based on Table 2.

As shown in Figure 1, metabolite profiling of the six plant families revealed that Aceraceae, Betulaceae, and Fagaceae clusters were separated from those of Rosaceae, Asteraceae, and Fabaceae. This was similar to the results of bioactivity analyses for the six families. Antioxidant and tyrosinase inhibition activity was averagely higher in Aceraceae, Betulaceae, and Fagaceae than in Asteraceae, Fabaceae, and Rosaceae (Figure 3). Moreover, most of the identified metabolites, such as isorhamnetin, quercetin, kaempferol, and their glycosides exhibited high concentrations in Betulaceae, Fagaceae, and Aceraceae (Figure 2A). Prior to this study, polyphenolic characterization of species in Betulaceae was performed, and isorhamnetin, quercetin, kaempferol, and their glycosides were identified as major flavonoids, which contribute to the anti-melano effect and antioxidant activity [40]. Furthermore, gallic acid and flavonoids were major polyphenols in plants in the Fagaceae family, which contribute to antioxidant activity [41,42]. Similar to the Betulaceae and Fagaceae, Aceraceae species were reported to exhibit antioxidant activity due to various phenolic compounds, such as quercetin, kaempferol, and flavonoid derivatives [43]. These results suggested that the relatively high contents of bioactive compounds in Aceraceae, Betulaceae, and Fagaceae might contribute to the high bioactivity of those families. For detailed study of correlation between metabolic differences and bioactivities of plant families, Fagaceae and Asteraceae were selected based on the results of the multivariate statistical analysis and bioactivities. Fagaceae was substantially separated from Asteraceae on the PLS-DA score plot (Figure 1A), and these two families clearly showed different antioxidant and tyrosinase inhibition activities (Figure 2B,D). To determine the significantly different metabolites between Fagaceae and Asteraceae, an orthogonal partial least squares discriminant analysis (OPLS-DA) was conducted (Figure 4). OPLS-DA is an extension of PLS-DA, which pursues the maximization of explained variance between groups. Fourteen metabolites were selected as discriminant metabolites between Fagaceae and Asteraceae by VIP > 0.7 and *p*-value < 0.05 (Table 2). Twelve metabolites were tentatively identified by comparing mass spectra and retention time of standard compounds, or mass to charge ratio, mass fragment patterns, and UV absorbance according to references [32,33,35]. Discriminant metabolites between Fagaceae and Asteraceae were indicated in a loading S-plot (Figure 4B). The relative contents of significantly different metabolites between Fagaceae and Asteraceae are visualized in the box and whisker plots (Figure S3). The contents of quinic acid (**1**), quercetin-3-*O*-arabinoside (**4**), quercetin-3-*O*-rhamnoside (**5**), kaempferol-3-*O*-rhamnoside (**7**), quercetin (**14**), kaempferol (**17**), 6-hydroxykaempferol-*O*-galloylhexoside (**3**), N.I. 4 (**20**), and quercetin-*O*-pentoside (**25**) were averagely higher in Fagaceae than in Asteraceae (Figure S3A). Various flavonoids and flavonoid glycosides were identified in Fagaceae, and the antioxidant activity of these compounds was reported [44].

**Figure 5:**
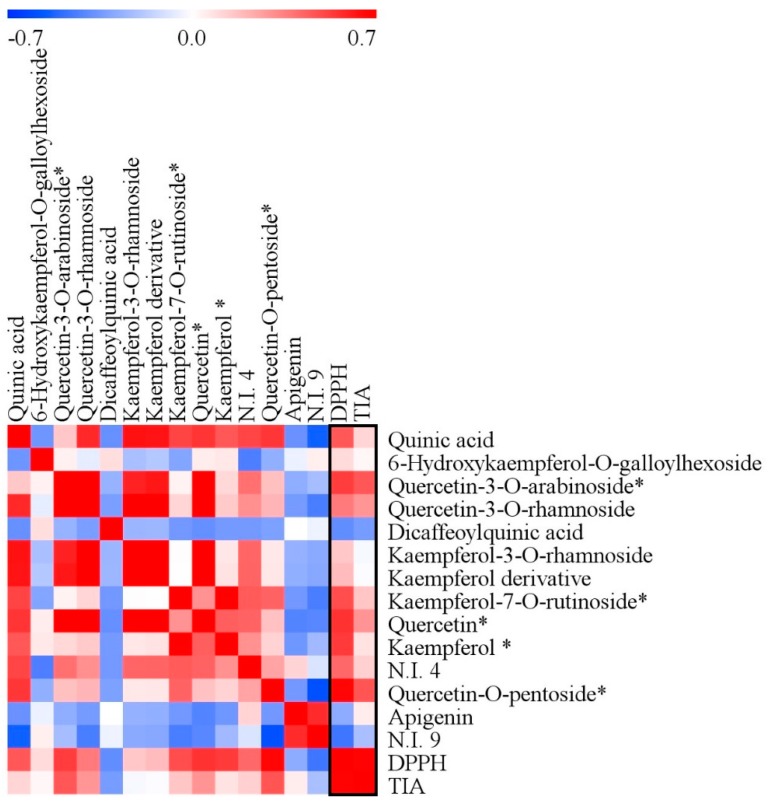
Correlation map of metabolites analyzed by ultrahigh performance liquid chromatography (UHPLC)-linear trap quadrupole-ion trap (LTQ-IT) mass spectrometry/mass spectrometry (MS/MS) with antioxidant and tyrosinase inhibition activities. Each square indicates *r* (Pearson’s correlation coefficient values for a pair of metabolites or antioxidant activity). The red color indicates positive (0 < *r* < 0.7) correlation and blue color indicates negative (−0.7 < *r* < 0) correlation. * The correlation coefficients of five metabolites are statistically significant at a level of 0.05 in bioactivities.

In order to visualize correlation of metabolites with antioxidant and tyrosinase inhibition activities, Pearson’s correlation test was used to construct a correlation map (Figure 5). Quinic acid (**1**), 6-hydroxykaempferol-*O*-galloylhexoside (**3**), quercetin-3-*O*-arabinoside (**4**), quercetin-3-*O*-rhamnoside (**5**), kaempferol-3-*O*-rhamnoside (**7**), kaempferol derivative (**8**), kaempferol-7-*O*-rutinoside (**13**), quercetin (**14**), kaempferol (**17**), N.I. 4 (**20**), and quercetin-*O*-pentoside (**25**) were positively correlated with antioxidant activity (0.02 < *r* < 0.47) and tyrosinase inhibition activity (0.10 < *r* < 0.67). There have been few studies regarding direct bioactivity of quinic acid, but quinic acid is a precursor of hydroxycinnamic acid derivatives, which have been reported to show antioxidant and tyrosinase inhibition activities [45]. Antioxidant and tyrosinase inhibition activities of quercetin, kaempferol, quercetin glycosides, and kaempferol glycosides have been reported [46]. These results suggested that the high bioactivities of Fagaceae could be related to the high contents of those metabolites because of their positive correlations with bioactivity. Dicaffeoylquinic acid (**6**), apigenin (**26**), and N.I. 9 (**27**) were negatively correlated with antioxidant (−0.47 < *r* < −0.29) and tyrosinase inhibition activities (−0.34 < *r* < −0.22). The results indicated that there were metabolic differences between Fagaceae and Asteraceae, and these metabolic differences contributed to different bioactivities of Fagaceae and Asteraceae. Similar to the findings in this study, Surveswaran *et al.* [47] reported that different contents of phenolic compounds and flavonoids affected different antioxidant capacities of plant species. Thus, the six plant families showed different metabolic states as demonstrated in the multivariate statistical analysis, and these contributed to different bioactivities among the six plant families.

**Table 2:** Significantly different metabolites between Fagaceae and Asteraceae analyzed by UHPLC-LTQ-IT-MS/MS.

No.	Putative Identification ^a^	RT ^b^ (min)	UHPLC-LTQXL-IT-MS/MS					*p*-Value	Id ^g^
			*m*/*z* Posi ^c^	*m*/*z* Nega ^d^	M.W. ^e^	MS^n^ Fragment Patterns ^f^	UV (nm)		
1	Quinic acid	1.02	-	191	192	173	214, 279	4.00E-04	Ref. [33]
3	6-Hydroxykaempferol-*O*-galloylhexoside	7.09	617	615	616	463 > 301	221, 265, 352	2.89E-02	Ref. [32]
4	Quercetin-3-*O*-arabinoside	7.89	435	433	434	301	239, 368	2.53E-02	Ref. [35]
5	Quercetin-3-*O*-rhamnoside	8.08	449	447	448	301 > 151	232, 277	4.70E-03	Ref. [35]
6	Dicaffeoylquinic acid	8.28	517	515	516	353 > 191	214, 322	4.76E-02	Ref. [33]
7	Kaempferol-3-*O*-rhamnoside	8.61	433	431	432	285	228, 280	3.95E-02	Ref. [35]
8	Kaempferol derivative	8.63	479	477	478	431 > 285	271, 281	3.63E-02	Ref. [35]
13	Kaempferol-7-*O*-rutinoside	9.34	595	593	594	447 > 285	276	9.00E-04	Ref. [33]
14	Quercetin	9.69	303	301	302	151	214, 274	9.00E-04	STD
17	Kaempferol	10.63	287	285	286		279	1.97E-02	STD
20	N.I. 4	11.96	309	307	308	289 > 235	214, 279	1.95E-02	
25	Quercetin-*O*-pentoside	7.83	435	433	434	301	217, 268	3.50E-03	Ref. [32]
26	Apigenin	10.47	271	269	270	269, 151	285, 318	4.01E-02	STD
27	N.I. 9	10.74	661	659	660		202, 280	3.20E-03	

^a^ Putative metabolites based on variable importance projection (VIP) analysis with cutoff value of 0.7 and a *p*-value <0.05. ^b^ Retention time. ^c^ Molecular ion detected in positive mode, [M + H]^+^. ^d^ Molecular ion detected in negative mode, [M − H]^−^. ^e^ Molecular weight. ^f^ MS^n^ fragment patterns detected in negative ion mode. ^g^ Identification: STD, standard compound/Ref., references.

## 3. Experimental Section

### 3.1. Chemicals and Reagents

Ethanol, methanol, acetonitrile, and water were purchased from Fisher Scientific (Pittsburgh, PA, USA). 2,2ʹʹ-Azinobis(3-ethylbenzothiazoline-6-sulfonic acid) diammonium salt (ABTS), 2,4,6-tripyridyl-s-triazine (TPTZ), hydrochloric acid (HCl), iron (III) chloride, acetic acid, 1,1-diphenyl-2-picrylhydrazyl (DPPH), 6-hydroxy-2,5,7,8-tetramethylchroman-2-carboxylic acid (Trolox), formic acid, gallic acid (purity, ≥98%), quercetin (purity, ≥95%), genistein (purity, ≥98%), and kaempferol (purity, ≥90%) were obtained from Sigma-Aldrich (St. Louis, MO, USA).

### 3.2. Plant Materials

Sixty-two indigenous Korean plant species (eight Aceraceae, eight Fagaceae, nine Asteraceae, nine Fabaceae, 10 Betulaceae, and 18 Rosaceae) were used in this study (Table 3). Plant samples were collected from seven provinces, two metropolitan cities, and one special self-governing province of Korea between May and October 2014. All voucher specimens were deposited in the herbarium of the National Institute of Biological Resources (NIBR, Incheon, Korea).

### 3.3. Sample Preparation

Plant samples were dried under shade, and each sample (100 g) was extracted three times with 70% ethanol (1000 mL). Each sample was concentrated by a rotary vacuum evaporator (Eyela, Tokyo, Japan) after filtration. The concentrated solution was freeze-dried and stored at below −70 °C before experimentation. Each extract sample (20 mg) was dissolved with 1 mL of 70% ethanol. Prior to ultrahigh performance liquid chromatography LTQ XL linear ion trap mass spectrometry/mass spectrometry (UHPLC-LTQ-XL-IT-MS/MS), 100 μL of each dissolved sample was filtered through a 0.2 μm PTFE filter.

### 3.4. UHPLC-LTQ-XL-IT-MS/MS Analysis

The Thermo Fischer Scientific LTQ XL linear ion trap mass spectrometry consisted of an electrospray interface (Thermo Fischer Scientific, San José, CA, USA) coupled with a DIONEX UltiMate 3000 RS Pump, RS Autosampler, RS Column Compartment, and RS Diode Array Detector (Dionex Corporation, Sunnyvale, CA, USA). Samples were separated on a Thermo Scientific Syncronis C18 UHPLC column with 1.7 μm particle size. The mobile phase consisted of A (0.1% (*v*/*v*) formic acid in water) and B (0.1% (*v*/*v*) formic acid in acetonitrile) and the gradient conditions were increased from 10% to 100% of solvent B over 18 min, and re-equilibrated to the initial condition for 4 min. The flow rate was 0.3 mL/min and the injection volume was 10 μL. Temperature of the column during measurement was maintained at 35 °C. The photodiode array was set at 200–600 nm for detection and managed by 3D field. Ion trap was performed in positive, negative, and full-scan ion modes within a range of 150–1000 *m*/*z*. The operating parameters were as follows: source voltage, ±5 kV, capillary voltage, 39 V; capillary temperature, 275 °C. Tandem MS analysis was performed by scan-type turbo data-dependent scanning (DDS) under the same conditions used for MS scanning.

**Table 3:** Information for samples used in this study.

No.	Family	Genus	Species	Collection Area	Collection Date
1	Aceraceae	*Acer*	*triflorum*	Sangjung-ri, Geumgwang-myeon, Anseong-si, Gyeonggi-do	2014-07-25
2	Aceraceae	*Acer*	*pictum*	Sangjung-ri, Geumgwang-myeon, Anseong-si, Gyeonggi-do	2014-07-25
3	Aceraceae	*Acer*	*buergerianum*	Janghyeon-ri, Cheongna-myeon, Boryeong-si, Chungcheongnam-do	2014-08-07
4	Aceraceae	*Acer*	*komarovii*	Gohan-ri, Gohan-eup, Jeongseon-gun, Gangwon-do	2014-08-30
5	Aceraceae	*Acer*	*tataricum*	Gurae-ri, Sangdong-eup, Yeongwol-gun, Gangwon-do	2014-08-30
6	Aceraceae	*Acer*	*pseudosieboldianum*	Gohan-ri, Gohan-eup, Jeongseon-gun, Gangwon-do	2014-08-30
7	Aceraceae	*Acer*	*pictum*	Jeodong-ri, Ulleung-eup, Ulleung-gun, Gyeongsangbuk-do	2014-07-16
8	Aceraceae	*Acer*	*palmatum*	Mamyeong-ri, Naechon-myeon, Pocheon-si, Gyeonggi-do	2014-08-08
9	Asteraceae	*Artemisia*	*capillaris*	Nadae-ri, Yaro-myeon, Hapcheon-gun, Gyeongsangnam-do	2014-08-21
10	Asteraceae	*Aster*	*pinnatifidus*	Geogi-ri, Jusang-myeon, Geochang-gun, Gyeongsangnam-do	2014-08-22
11	Asteraceae	*Bidens*	*bipinnata*	Dongmak-ri, Yeoncheon-eup, Yeoncheon-gun, Gyeonggi-do	2014-08-19
12	Asteraceae	*Conyza*	*canadensis*	Sangdodae-ri, Sangchon-myeon, Yeongdong-gun, Chungcheongbuk-do	2014-08-14
13	Asteraceae	*Erigeron*	*annuus*	Dongmak-ri, Yeoncheon-eup, Yeoncheon-gun, Gyeonggi-do	2014-08-05
14	Asteraceae	*Helianthus*	*tuberosus*	Jiro-ri, Byeongyeong-myeon, Gangjin-gun, Jeollanam-do	2014-08-12
15	Asteraceae	*Lactuca*	*indica*	Gomo-ri, Soheul-eup, Pocheon-si, Gyeonggi-do	2014-08-24
16	Asteraceae	*Saussurea*	*pulchella*	Gohan-ri, Gohan-eup, Jeongseon-gun, Gangwon-do	2014-08-30
17	Asteraceae	*Sigesbeckia*	*pubescens*	Gurae-ri, Sangdong-eup, Yeongwol-gun, Gangwon-do	2014-08-30
18	Fabaceae	*Albizia*	*julibrissin*	Daechi-ri, Daechi-myeon, Cheongyang-gun, Chungcheongnam-do	2014-08-06
19	Fabaceae	*Desmodium*	*caudatum*	Seonheul-ri, Jocheon-eup, Jeju-si, Jeju special self-governing province	2014-08-24
20	Fabaceae	*Lespedeza*	*bicolor*	Sin-ri, Goryeong-eup, Goryeong-gun, Gyeongsangbuk-do	2014-07-23
21	Fabaceae	*Lespedeza*	*cuneata*	Geogi-ri, Jusang-myeon, Geochang-gun, Gyeongsangnam-do	2014-08-22
22	Fabaceae	*Lespedeza*	*maximowiczii*	Gohan-ri, Gohan-eup, Jeongseon-gun, Gangwon-do	2014-08-30
23	Fabaceae	*Pueraria*	*lobata*	Mamyeong-ri, Naechon-myeon, Pocheon-si, Gyeonggi-do	2014-08-04
24	Fabaceae	*Robinia*	*pseudoacacia*	Sin-ri, Goryeong-eup, Goryeong-gun, Gyeongsangbuk-do	2014-10-23
25	Fabaceae	*Sophora*	*flavescens*	Hanggok-ri, Gunbuk-myeon, Okcheon-gun, Chungcheongbuk-do	2014-08-18
26	Fabaceae	*Sophora*	*japonica*	Geogi-ri, Jusang-myeon, Geochang-gun, Gyeongsangnam-do	2014-08-22
27	Fagaceae	*Castanea*	*crenata*	Hasong-ri, Hwaseo-myeon, Sangju-si, Gyeongsangbuk-do	2014-07-27
28	Fagaceae	*Castanopsis*	*sieboldii*	Hannam-ri, Namwon-eup, Seogwipo-si, Jeju special self-governing province	2014-08-25
29	Fagaceae	*Fagus*	*engleriana*	Sadong-ri, Ulleung-eup, Ulleung-gun, Gyeongsangbuk-do	2014-07-16
30	Fagaceae	*Quercus*	*mongolica*	Hasong-ri, Hwaseo-myeon, Sangju-si, Gyeongsangbuk-do	2014-07-27
31	Fagaceae	*Quercus*	*variabilis*	Daechi-ri, Daechi-myeon, Cheongyang-gun, Chungcheongnam-do	2014-08-06
32	Fagaceae	*Quercus*	*acuta*	Hannam-ri, Namwon-eup, Seogwipo-si, Jeju special self-governing province	2014-08-25
33	Fagaceae	*Quercus*	*aliena*	Daechi-ri, Daechi-myeon, Cheongyang-gun, Chungcheongnam-do	2014-09-19
34	Fagaceae	*Quercus*	*serrata*	Mamyeong-ri, Naechon-myeon, Pocheon-si, Gyeonggi-do	2014-08-05
35	Betulaceae	*Alnus*	*firma*	Sin-ri, Goryeong-eup, Goryeong-gun, Gyeongsangbuk-do	2014-07-23
36	Betulaceae	*Alnus*	*hirsuta*	Sangjung-ri, Geumgwang-myeon, Anseong-si, Gyeonggi-do	2014-07-25
37	Betulaceae	*Alnus*	*japonica*	Yonggi-ri, Gibuk-myeon, Buk-gu, Pohang-si, Gyeongsangbuk-do	2014-07-30
38	Betulaceae	*Betula*	*schmidtii*	Icheon-ri, Sangbuk-myeon, Ulju-gun, Ulsan	2014-08-01
39	Betulaceae	*Betula*	*dahurica*	Ungyo-ri, Bangnim-myeon, Pyeongchang-gun, Gangwon-do	2014-08-08
40	Betulaceae	*Betula*	*pendula*	Sogye-ri, Hwanggan-myeon, Yeongdong-gun, Chungcheongbuk-do	2014-08-14
41	Betulaceae	*Carpinus*	*cordata*	Apgok-ri, Bongsan-myeon, Hapcheon-gun, Gyeongsangnam-do	2014-07-24
42	Betulaceae	*Carpinus*	*turczaninowii*	Jiro-ri, Byeongyeong-myeon, Gangjin-gun, Jeollanam-do	2014-08-12
43	Betulaceae	*Carpinus*	*laxiflora*	Seonheul-ri, Jocheon-eup, Jeju-si, Jeju special self-governing province	2014-08-24
44	Betulaceae	*Corylus*	*heterophylla*	Apgok-ri, Bongsan-myeon, Hapcheon-gun, Gyeongsangnam-do	2014-07-24
45	Rosaceae	*Chaenomeles*	*sinensis*	Ojeong-dong, Daedeok-gu, Daejeon	2014-08-10
46	Rosaceae	*Crataegus*	*pinnatifida*	Gurae-ri, Sangdong-eup, Yeongwol-gun, Gangwon-do	2014-08-30
47	Rosaceae	*Eriobotrya*	*japonica*	Jiro-ri, Byeongyeong-myeon, Gangjin-gun, Jeollanam-do	2014-08-13
48	Rosaceae	*Pourthiaea*	*villosa*	Seonheul-ri, Jocheon-eup, Jeju-si, Jeju special self-governing province	2014-08-24
49	Rosaceae	*Prunus*	*armeniaca*	Ojeong-dong, Daedeok-gu, Daejeon	2014-07-20
50	Rosaceae	*Prunus*	*yedoensis*	Janghyeon-ri, Cheongna-myeon, Boryeong-si, Chungcheongnam-do	2014-08-07
51	Rosaceae	*Prunus*	*maackii*	Gurae-ri, Sangdong-eup, Yeongwol-gun, Gangwon-do	2014-08-30
52	Rosaceae	*Prunus*	*padus*	Gohan-ri, Gohan-eup, Jeongseon-gun, Gangwon-do	2014-05-22
53	Rosaceae	*Prunus*	*sp.*	Gomo-ri, Soheul-eup, Pocheon-si, Gyeonggi-do	2014-08-08
54	Rosaceae	*Pyrus*	*ussuriensis*	Icheon-ri, Sangbuk-myeon, Ulju-gun, Ulsan	2014-08-01
55	Rosaceae	*Rosa*	*multiflora*	Nadae-ri, Yaro-myeon, Hapcheon-gun, Gyeongsangnam-do	2014-08-21
56	Rosaceae	*Rubus*	*coreanus*	Sogye-ri, Hwanggan-myeon, Yeongdong-gun, Chungcheongbuk-do	2014-08-14
57	Rosaceae	*Rubus*	*crataegifolius*	Nadae-ri, Yaro-myeon, Hapcheon-gun, Gyeongsangnam-do	2014-08-21
58	Rosaceae	*Rubus*	*phoenicolasius*	Nadae-ri, Yaro-myeon, Hapcheon-gun, Gyeongsangnam-do	2014-08-21
59	Rosaceae	*Sanguisorba*	*officinalis*	Nadae-ri, Yaro-myeon, Hapcheon-gun, Gyeongsangnam-do	2014-08-21
60	Rosaceae	*Sorbus*	*commixta*	Jeodong-ri, Ulleung-eup, Ulleung-gun, Gyeongsangbuk-do	2014-07-16
61	Rosaceae	*Spiraea*	*prunifolia*	Ungyo-ri, Bangnim-myeon, Pyeongchang-gun, Gangwon-do	2014-08-08
62	Rosaceae	*Spiraea*	*salicifolia*	Ungyo-ri, Bangnim-myeon, Pyeongchang-gun, Gangwon-do	2014-08-08

### 3.5. Data Processing and Statistical Analysis

The UHPLC-LTQ-IT-MS/MS data were acquired with Xcalibar software (version 2.00, Thermo Fischer Scientific), and raw data files were converted to NetCDF (*.cdf) format using Xcalibar software. After conversion, the NetCDF files were subjected to preprocessing, correction of retention time and baseline, and peak extraction using the MetAlign software package [48]. The resulting data were exported to Microsoft Excel (Microsoft, Redmond, WA, USA). Multivariate statistical analysis was processed using SIMCA-P + 12.0 software (Umetrics, Umea, Sweden). Principal component analysis (PCA), partial least-square discriminant analysis (PLS-DA), orthogonal partial least-square discriminant analysis (OPLS-DA), and S-plots were performed to determine metabolite differences between plant species. The variables were selected based on variable importance in the projection (VIP) value and significant differences were determined by analysis of variance (ANOVA). Box-whisker plots were performed using STATISTICA (version 7.0, StatSoft Inc., Tulsa, OK, USA). SPSS for Windows (version 12.0; SPSS, Inc., Chicago, IL, USA) was used to calculate Pearson’s correlation coefficient between metabolites and bioactivity assays. After multivariate statistical analysis, significantly different metabolites were positively identified using standard compounds by comparing both mass spectra and retention time. When standard compounds were not available, a tentative identification was performed based on the MS spectra using the NIST05 MS Library (NIST, 2005), combined chemical dictionary version 7.2 (Chapman and Hall/CRC), and references.

### 3.6. Bioactivity Assays

For antioxidant activity by DPPH free radical scavenging assay, we followed Lee *et al.* [49] with some modifications. Reaction mixtures containing 20 μL of each dissolved sample and 180 μL of DPPH ethanol solution (0.2 mM) were incubated at room temperature for 20 min in 96-well plates. The absorbance of the DPPH free radicals was measured at 515 nm using a microplate reader. Results were expressed in mg of trolox equivalent concentration. Trolox standard solutions were serially diluted from 1 mM to 0.0625 mM. Experiments were carried out in triplicate*.*

Mushroom tyrosinase inhibition assay was carried out according to Kim *et al.* [50] with some variations. Reaction mixtures containing 153 μL of 0.1 M sodium phosphate buffer (pH 6.5), 36 μL of 1.5 mM l-tyrosine in 0.1 M sodium phosphate buffer (pH 6.5), 6 μL of mushroom tyrosinase (2500 unit/mL), and 5 μL of each dissolved sample were incubated at 37 °C for 20 min and the absorbance was measured at 490 nm using a microplate reader. Kojic acid was used as a positive control and 100% methanol was used as the negative control. Experiments were conducted in triplicate. Tyrosinase inhibition activity was calculated as follows:

Tyrosinase inhibition activity (%) = [(C_20min_ − C_0min_) − (S_20min_ − S_0min_)]/(C_20min_ − C_0min_) × 100 (1)

where C_20min_ is the absorbance of the negative control after 20 min, C_0min_ is the absorbance of the negative control at 0 min, S_20min_ is the absorbance of the sample at 20 min, and S_0min_ is the absorbance of the sample at 0 min.

## 4. Conclusions

Chemotaxonomic metabolite profiling of 62 indigenous Korean plant species was performed by UHPLC-LTQ-IT-MS/MS combined with multivariate statistical analysis. Both PLS-DA score plots and HCA dendrograms showed that the 62 species were clearly separated according to family. In particular, Aceraceae, Betulaceae, and Fagaceae were distinguished from Rosaceae, Fabaceae, and Asteraceae. Quinic acid, gallic acid, digalloyl-hexoside, quercetin, kaempferol, isorhamnetin, quercetin derivatives, kaempferol derivatives, patuletin, dicaffeoylquinic acid, catechin, genistein, 6-hydroxykaempferol-*O*-galloylhexoside, and eight non-identified metabolites were found to be the major metabolites separating families, and their relative concentrations were compared. Antioxidant activity and tyrosinase inhibition activity were high in Aceraceae, Fagaceae, and Betulaceae. Fagaceae and Asteraceae were selected based on results of PLS-DA and bioactivities for correlation between metabolites and bioactivities. Among significantly different metabolites, quinic acid, quercetin, kaempferol, quercetin derivatives, kaempferol derivatives, and 1 non-identified metabolite had high concentrations in Fagaceae and these metabolites were positively correlated with antioxidant and tyrosinase inhibition activities. These results indicated that high concentrations of these metabolites existing in Fagaceae contributed to antioxidant and tyrosinase inhibition activities. In summary, this study suggested that metabolomics-based metabolite profiling was useful for chemotaxonomic analysis in various plant species, and could be helpful to select useful plant resources and identify beneficial phytochemicals.

## Supplementary Materials

Supplementary materials can be accessed at: http://www.mdpi.com/1420-3049/20/11/19652/s1.
