# Supplementary material for: Chemotaxonomic Metabolite Profiling of 62 Indigenous Plant Species and Its Correlation with Bioactivities
Source: Molecules. 2015 Nov 2;20(11):19719–34. doi: 10.3390/molecules201119652 (PMC6332367; doi:10.3390/molecules201119652)
Supplement: Supplementary file 1 [file molecules-20-19652-s001.pdf]

molecules

MDPI

Supplementary Informations

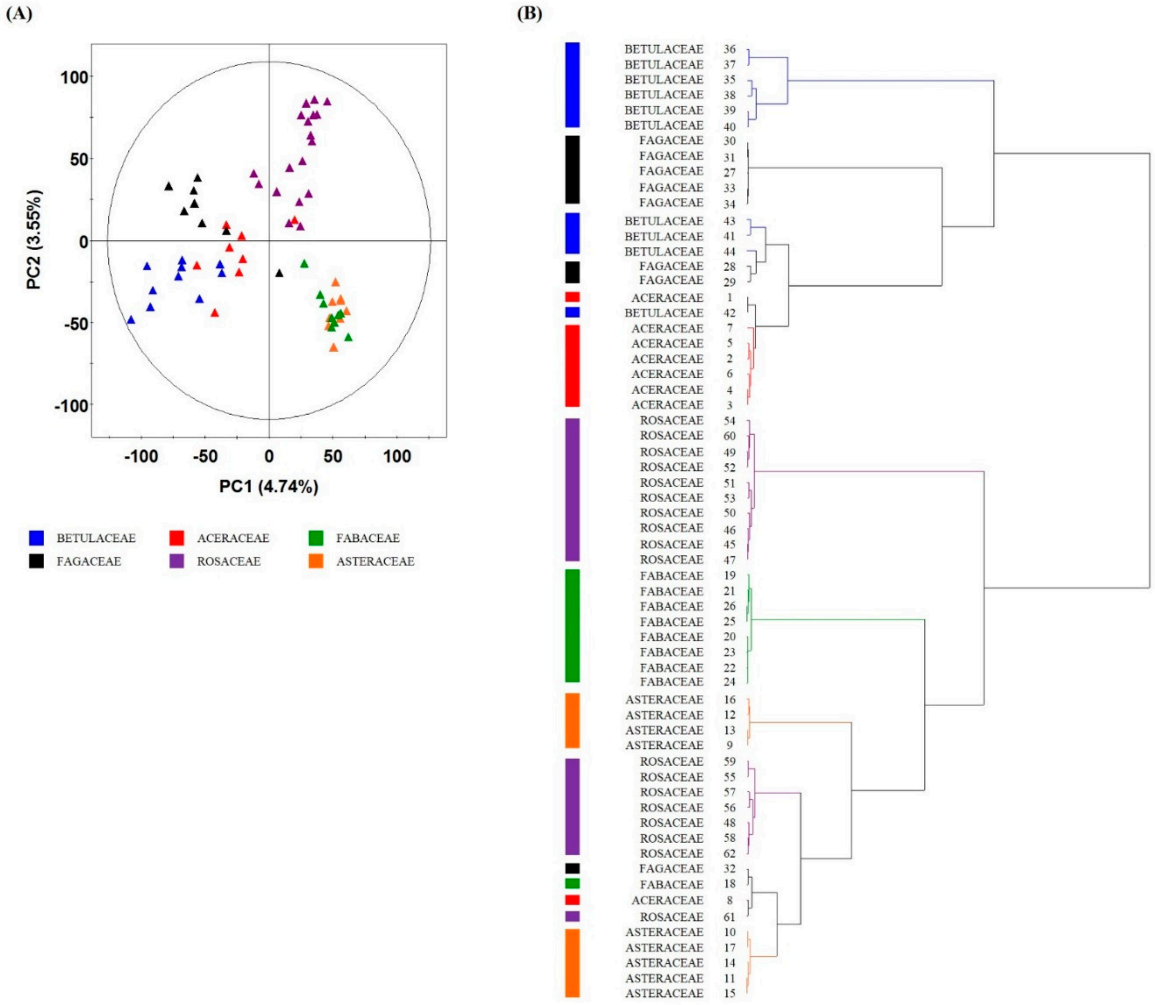

Figure S1. Principal component analysis (PCA) score plot (**A**) and hierarchical cluster analysis (HCA) dendrogram based on PCA results (**B**) derived from the ultrahigh performance liquid chromatography (UHPLC)-linear trap quadrupole-ion trap (LTQ-IT) mass spectrometry/mass spectrometry (MS/MS) data of 62 indigenous Korean plant species. Samples are colored according to family.

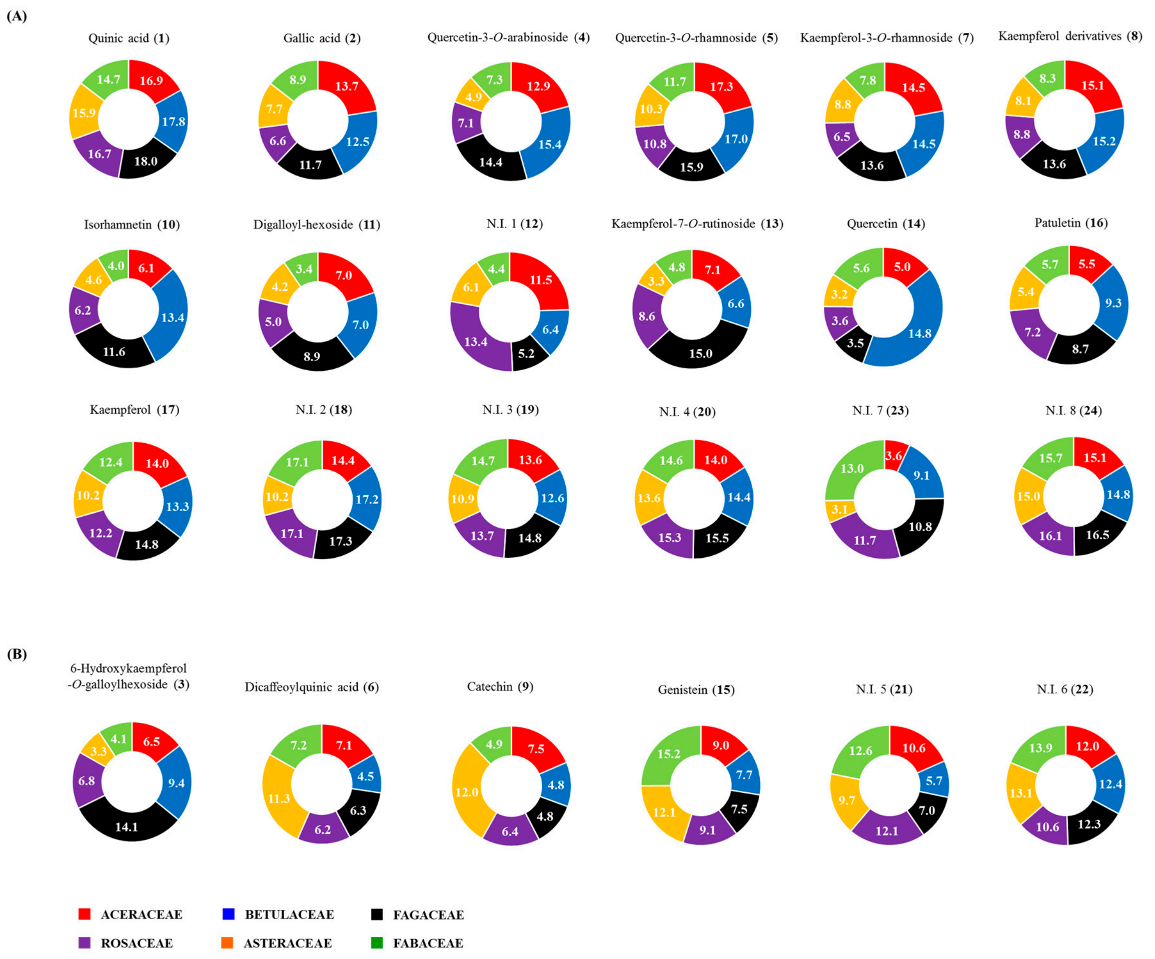

Figure S2. Doughnut charts of an average percentage of individual compounds in plant families. Levels of compounds are peak area transformed by $\log_{10}$ analyzed by ultrahigh performance liquid chromatography (UHPLC)-linear trap quadrupole-ion trap (LTQ-IT) mass spectrometry/mass spectrometry (MS/MS).

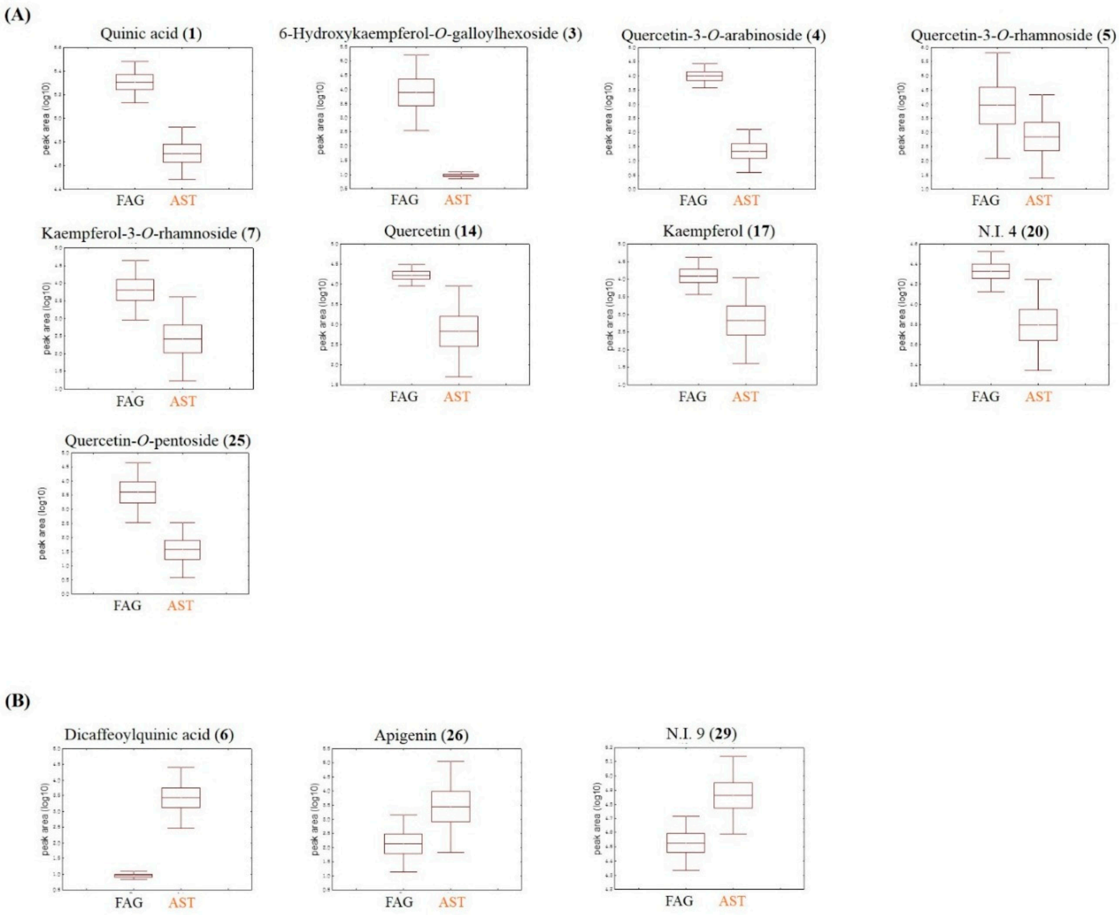

Figure S3. Box and whisker plots of significantly different metabolites between Fagaceae and Asteraceae analyzed by ultrahigh performance liquid chromatography (UHPLC)-linear trap quadrupole-ion trap (LTQ-IT) mass spectrometry/mass spectrometry (MS/MS). Metabolites higher in Fagaceae than in Asteraceae (**A**) and metabolites higher in Asteraceae than in Fagaceae (**B**) are expressed, respectively. The *Y*-axis of box and whisker plots indicates the peak area of metabolites transformed by $\log_{10}$ (FAG, Fagaceae; AST, Asteraceae; *Line*, mean; *box*, standard error; *whisker*, standard deviation).
